# On the influence of interface charging dynamics and stressing conditions in strained silicon devices

**DOI:** 10.1038/s41598-017-05067-9

**Published:** 2017-08-03

**Authors:** Irene Olivares, Todora Angelova, Pablo Sanchis

**Affiliations:** Nanophotonics Technology Center, Universitat Politècnica València, Camino de Vera s/n, Valencia, 46022 Spain

## Abstract

The performance of strained silicon devices based on the deposition of a top silicon nitride layer with high stress have been thoroughly analyzed by means of simulations and experimental results. Results clearly indicate that the electro-optic static response is basically governed by carrier effects. A first evidence is the appearance of a variable optical absorption with the applied voltage that should not occur in case of having a purely electro-optic Pockels effect. However, hysteresis and saturation effects are also observed. We demonstrate that such effects are mainly due to the carrier trapping dynamics at the interface between the silicon and the silicon nitride and their influence on the silicon nitride charge. This theory is further confirmed by analyzing identical devices but with the silicon nitride cladding layer optimized to have intrinsic stresses of opposite sign and magnitude. The latter is achieved by a post annealing process which produces a defect healing and consequently a reduction of the silicon nitride charge. Raman measurements are also carried out to confirm the obtained results.

## Introduction

The silicon platform has the unique capability for enabling a monolithic integration of photonic and electronic circuits with a low cost standardized fabrication process. However, the centrosymmetric crystalline structure of silicon prevents the development of key photonic components such as electro-optic modulators due to the absence of the second-order non linearity. The main approach to overcome the intrinsic limitations of silicon has relayed on the plasma dispersion effect, which is currently the most effective mechanism for changing the silicon refractive index at a fast rate^1^. However, a trade off between low driving voltages, high bandwidth and low losses is usually given in part due to the optical absorption inherent to the plasma dispersion effect.

High modulation speeds can be achieved by means of the Pockels effect without penalizing insertion losses. Therefore, different approaches are being followed for trying to have access to such feature in the silicon platform. The integration on silicon of ferroelectric materials with high Pockels coefficients, such as LiNbO_3_^2^ or BaTiO_3_^3, 4^ is currently one of the main approaches. The use of nonlinear polymers with a high second-order nonlinearity has also been proposed^5^. However, the demonstration of Pockels effect in strained silicon by Jacobsen *et al*.^6^ opened the door to a new route for a CMOS compatible integration of fast and low loss electro-optic modulators at minimum complexity and cost. Since then a large number of works have been reported to analyze and optimize the Pockels effect in strained silicon devices^7–18^ and values above *χ* ^(2)^ = 100 pm/V have been theoretically proven^19^. In the last years, discrepancies between theoretical and experimental results have made relevant that other effects could also take place in the measured responses^20^. Most recently, the influence of carrier effects have been demonstrated to play a prominent role in the electro-optic response^21–24^. High frequency measurements have shown a modulation response vanishing for speeds much faster than the effective carrier lifetime^21^. The free carrier distribution inside the silicon waveguide depends on the fixed charge of the cladding layer^22^. Variations of the carrier distribution can affect the electric field inside the waveguide and therefore the modulation induced by the Pockels effect. However, an electro-optic response induced by the free carrier distribution has been demonstrated by using cladding materials with different and opposite fixed charge concentrations and interface traps densities^23^. Furthermore, the injection of free carriers in the silicon waveguide in response to an applied electric field has also been proposed as a possible mechanism responsible of the electro-optic response^24^.

In this work, the influence of interface charging dynamics and stressing conditions on the electro-optic response of strained silicon devices is discussed. The paper has been divided in two main parts. In the first part, the impact of the charge interchange at the interface and the silicon nitride charge are analyzed by means of simulations and experimental results. Secondly, the influence of stress is analyzed not only as a function of magnitude but also for different types of stress by fabricating samples with a silicon nitride layer with both compressive and tensile intrinsic stress. Furthermore, an additional annealing step, which changes the intrinsic stress of the silicon nitride, is carried out to further investigate its influence. Results provide an additional confirmation of the strong contribution of carrier effects taking place in the electro-optic response.

## Results and Discussion

**Figure 1:**
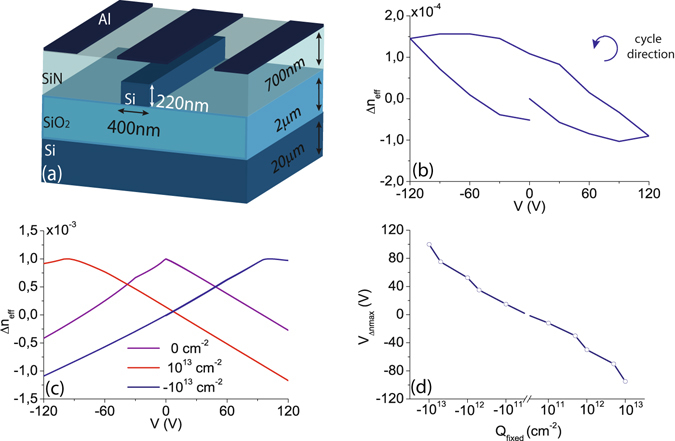
Free carrier simulation results and experimental effective index change. (**a**) Sketch of the simulated device, (**b**) experimental effective index change for the tensile stress sample, (**c**) effective index change obtained by simulations for different fixed charge concentrations and (**d**) voltage at which the index curve is centered as a function of the fixed charge.

An asymmetric Mach Zehnder interferometer (MZI) with a length difference between arms of 180 *μ*m has been used to analyze the electro-optic performance. The waveguide structure has been designed to maximize the strain inside the core taking into account a SOI substrate with a top silicon layer of 220 nm thickness^25^. A full etch depth and a waveguide width of 400 nm have been chosen to increase the stress at the walls. Furthermore, the thickness of the silicon nitride layer has been optimized to 700 nm. Alumnium electrodes have been placed on top of the silicon nitride to achieve an active length of 1 mm. A sketch of the waveguide structure is shown in Fig. 1(a). The silicon nitride has been deposited by means of PECVD and process parameters have been changed to control the intrinsic film stress. A compressive stress as high as −2 GPa and a maximum tensile stress of 419 MPa have been achieved by varying temperature and the concentration ratio between silane and ammonia. Therefore, two identical samples but with opposite silicon nitride intrinsic stress have been fabricated.

### Influence of free carriers and interface traps

The sample with tensile stress has been firstly characterized. The change of the effective refractive index as a function of the applied voltage is shown in Fig. 1(b). It can be seen that there is hysteresis and a saturation effect. The saturation can be observed starting at around −30 V to higher negative values, where the index change remains rather constant despite the increase in voltage. Simulations have been carried out to analyze the potential impact of the free carriers redistribution inside the slightly p-doped silicon waveguide on the measured response.

The simulated effective index change as a function of the applied voltage is shown in Fig. 1(c) for different fixed charge concentrations at the silicon-silicon nitride interface. As it was pointed out by Azadeh *et al*.^22^, a fixed charge stored in the silicon nitride film causes a displacement of the index curve, which may give rise to a linear response in a certain range of applied voltages. Figure 1(d) shows the voltage at which the index curve is centered depending on the fixed charge. Typical values reported for silicon nitride are on the order of 1–5·10^12^ *cm* ^−2^ so that simulations have been carried out in the range [−10^13^, 10^13^] *cm* ^−2^ to take into account slightly high densities. The linear relationship of the experimental response with the applied voltage would be in agreement with simulations in case that the positive fixed charge stored within the silicon nitride were around 10^13^ *cm* ^−2^. However, a discrepancy of around one order of magnitude is found between the simulated and the experimental effective refractive index change. The maximum effective index change in simulations is in the order of 10^−3^ while experimentally only reaches 10^−4^. The origin of this discrepancy is attributed to a charge interchange at the interface between silicon and silicon nitride, which would also explain the presence of the hysteresis and saturation effects.

**Figure 2:**
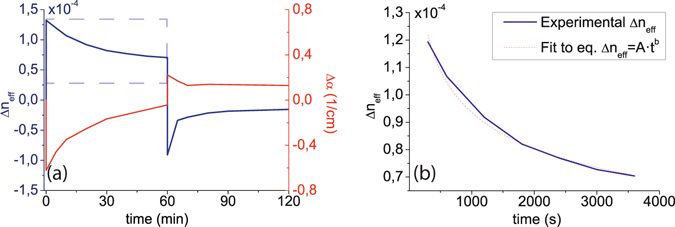
Experimental effective index change and absorption time evolution. (**a**) Time evolution of the absorption and effective index change for the soaking experiments. At the high voltage phase, shown in a dashed square, voltage was kept constant at −120 V during the first 60 minutes. The recovery phase at 0 V was monitored during the subsequent hour. (**b**) Effective index fit to the theoretical time dependency of Eq. (1) for the high voltage phase.

The trapping properties of silicon nitride films have been extensively studied in the microelectronics industry. The material is known due to its high defect concentration, positive fixed charge and ability to trap carriers from the silicon substrate in MOSFET devices^26, 27^. Although charge in the bulk or away from the interface is actually fixed, there is thought to be a part of it that can be interchanged between silicon and silicon nitride. The amount of fixed charge is usually studied using metal-insulator-semiconductor (MIS) structures. The charge of defects and dangling bonds can be altered at the interface or near the interface by applying a bias, causing the trapping and detrapping of carriers and resulting in a varying fixed charge, Q_*fixed*_^28^, and hysteresis in the C-V measurement. Furthermore, the creation of new interface traps is well documented due to processes such as carrier injection into the insulator film (HCI) and bias temperature instability (BTI). When high voltages are applied to the gate, high carrier concentrations are accumulated next to the interface and Si-H, N-H bonds can be broken mediated by a carrier trapping mechanism, giving rise to a great amount of new interface traps^29–31^. Both are slow processes involving the release and diffusion of hydrogen at the interface and usually take place in a range from less than seconds to minutes and hours. Since our measurement time is around two minutes for each measured voltage, those processes can be taking place when we perform the hysteresis cycle. In order to further investigate if HCI or BTI processes are present in our devices and, therefore, new interface traps are being created, soaking measurements have been carried out. Experiments performed in MIS structures usually consist in a constant gate applied voltage during a large period of time and a relax phase at 0 V during a similar range of time. The same procedure has been applied to our devices, maintaining a voltage of −120 V during one hour and monitoring the resonance shift of the MZI response. The measured variation of the effective index with time is depicted in Fig. 2(a). It can be seen that the index change decays with time at almost half of its initial value. The rate at which traps are being created is usually characterized with a time dependency in the form

1 $${\rm{\Delta }}{N}_{it}=A{t}^{n}$$

where n is typically in the range of 0.2–0.3 or around 0.5 respectively for negative BTI (NBTI) and HCI^29–31^. The effective index variation when the high voltage is applied has been fitted to an analogous time dependency to confirm its presence. The result is depicted in Fig. 2(b), showing an almost perfect fit (*R* ^2^ = 0.992) and obtaining a value of n = 0.2197 for the exponent, which would agree quite well with the theory of a NBTI process. The behavior of the optical absorption has also been characterized by means of a straight waveguide fabricated in the same sample. An electrode equal to that used on the MZI was located on top of the waveguide and an analogous measurement procedure was carried out. Figure 2(a) shows also the evolution of the absorption with time. Absorption values are negative indicating that they are lower compared to the one at 0 V and increase with time until the voltage of −120 V is no longer applied. This behavior is also in agreement with the creation of new interface traps. The trapping of more carriers gradually increases the silicon nitride fixed charge and consequently the absorption becomes higher with time while the effective index change decays.

**Figure 3:**
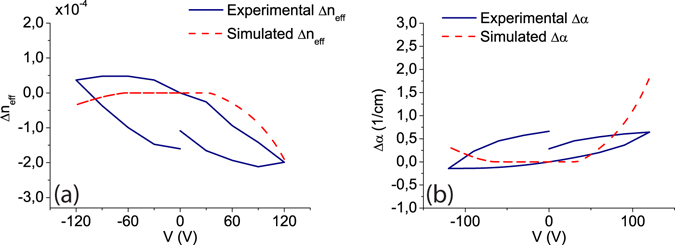
Simulation and experimental results comparison. The simulated (**a**) refractive index change and (**b**) absorption have been obtained taking into account the interface traps parameters depicted in Table 1.

The influence of interface traps on the electro-optic response has been analyzed by means of simulations. A continuous interface trap density distribution in energy is usually found within the silicon band gap, which is commonly characterized by means of deep level transient spectroscopy (DLTS) using MIS structures. Values around 1–5·10^11^ *cm* ^−2^/eV are usually obtained at midgap^32, 33^ but it should be noticed that the minimum interface trap density is generally found for stoichiometric or near stoichiometric silicon nitride films^33^. However, our samples have a nitride to silicon ratio [N]/[Si] of 1.5 and 1.7 for the tensile and compressive samples, respectively, far from the stoichiometric one (1.33). Therefore, the trap density could be higher. Furthermore, an increase of several orders of magnitude of interface traps^33^ and stored silicon nitride charge^28^ are measured when high voltages are applied in MIS structures, reaching charge densities even above 1·10^13^ *cm* ^−2^. Taking that into account, the interface trap parameters shown in Table 1, with typical capture cross sections reported in the literature^32^, and a fixed charge of 8·10^12^ *cm* ^−2^ were considered in the simulations. In such a way, simulated values very close to those experimentally observed have been obtained, as it can be seen in Fig. 3. Both, the experimental and simulated effective index change (Fig. 3(a)) and optical absorption (Fig. 3(b)) are in the same order of magnitude. Furthermore, although it was not possible to include the time dependency and so the hysteresis effect is not present in the simulations, the variation with the applied voltage is also in good agreement, thus supporting the influence and presence of interface states at the silicon-silicon nitride interface. Due to the fact that hysteresis has not been included, the only part of the experimental curve that could be simulated is the continuous one from 120 V to −120 V. Therefore, in Fig. 3, the effective index change and absorption are normalized to the corresponding values at 0 V of this part of the hysteresis cycle.

**Table 1:** Values of the interface trap parameters for the acceptor and donor states included in the simulations shown in Fig. 3.

Energy level	Density (*cm* ^−2^)	*σ*_*n*_ (*cm* ^−2^)	*σ*_*p*_ (*cm* ^−2^)	Energy level	Density (*cm* ^−2^)	*σ*_*n*_ (*cm* ^−2^)	*σ*_*p*_ (*cm* ^−2^)
*E*_*trap*_ − *E*_*v*_ (eV)	Donor states			*E*_*trap*_ − *E*_*v*_ (eV)	Acceptor states		
0.6	6·10^12^	10^−18^	10^−18^	0.2	8·10^12^	10^−18^	10^−18^
0.4	7·10^12^	10^−13^	10^−13^	0.4	7·10^12^	10^−13^	10^−13^
0.2	8·10^12^	10^−11^	10^−11^	0.6	6·10^12^	10^−11^	10^−11^

### Annealing study

**Figure 4:**
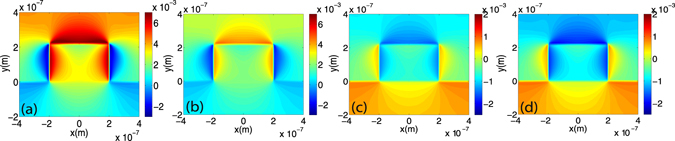
Effect of the annealing on the strain inside the structures. Simulated *ε*_*yy*_ strain component for: the compressive stress sample (**a**) before and (**b**) after the annealing process, tensile stress sample (**c**) before and (**d**) after the annealing process. The annealing was performed at 500 °C during 30 minutes in atmospheric environment.

**Figure 5:**
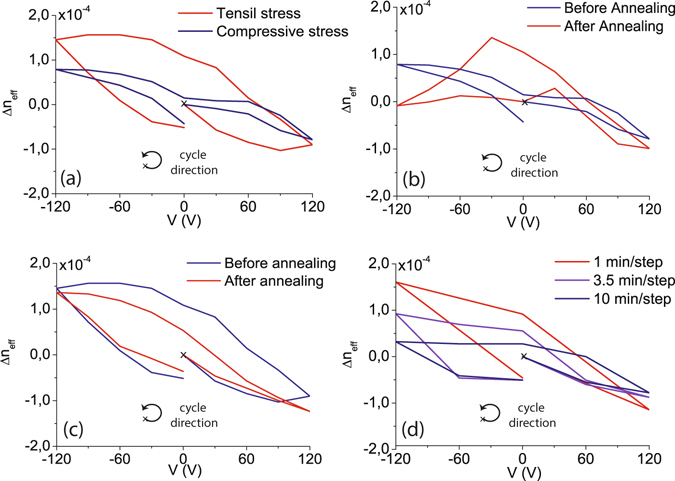
Experimental results for the compressive and tensile stress samples under different annealing and time measurement conditions. (**a**) Experimental refractive index change for the compressive and tensile samples before annealing. Comparison of results before and after the annealing for (**b**) the compressive and (**c**) tensile samples. (**d**) Results for the sample with tensile stress after annealing as a function of different times steps per each voltage measurement. The annealing process was performed at 500 °C during 30 minutes in atmospheric environment.

In order to further investigate how a change in stress affects the electro-optic performance, a subsequent annealing step was applied to the samples with both compressive and tensile stress making possible to study the same structures with different stress magnitudes. Samples were heated at 500 °C during 30 min in atmospheric environment. The measured intrinsic stress after annealing was −1.25 GPa and 530 MPa for the compressive and tensile samples respectively. Simulations have been carried out to evaluate the induced strain inside the waveguide structure. Figure 4(a) shows the *ε*_*yy*_ strain component for the compressive sample before annealing and Fig. 4(b) after annealing. Analogously, Fig. 4(c) and (d) show the results for the tensile sample before and after annealing respectively. Pockels effect should arise as a result of the silicon lattice asymmetric deformation. Therefore, as a way of evaluating the impact of the change in stress, the overlap between the strain gradient and the TE fundamental mode has been calculated using the following figure of merit^13, 25^:

2 $$FOM=\frac{{\iint }_{WG}{|E|}^{2}(|\frac{\partial {\varepsilon }_{xx}}{\partial x}|+|\frac{\partial {\varepsilon }_{xx}}{\partial y}|+|\frac{\partial {\varepsilon }_{yy}}{\partial x}|+|\frac{\partial {\varepsilon }_{yy}}{\partial y}|)dxdy}{\iint {|E|}^{2}dxdy}$$

where *E* refers to the electric field of the optical mode and *ε*_*xx*_, *ε*_*yy*_ are the main strain components. The annealing process has opposite effects for the tensile and compressive cases. While in the first one the stress is clearly increased and the FOM is improved $(\frac{FO{M}_{afterannealing}\,\mathrm{(530}MPa)}{FO{M}_{beforeannealing}\,\mathrm{(419}MPa)}\approx 1.24)$, the overlap integral is decreased in the second case $(\frac{FO{M}_{afterannealing}\,(-1.25GPa)}{FO{M}_{beforeannealing}\,(-2GPa)}\approx 0.62)$. Therefore, if the Pockels effect would play a significant role, these changes should be reflected in the electro-optic performance. The effective index change as a function of the applied voltage is shown in Fig. 5(a) for both compressive and tensile stress samples before the annealing process. It can be observed that despite the large difference not only in the sign of the applied stress but also in magnitude, a similar effective index change is found on the order of 10^−4^. Furthermore, although the compressive stress is around five times more intense than the tensile stress, $\frac{FOM(-2GPa)}{FOM\mathrm{(419}MPa)}=4.5$, the effective index change is even slightly higher for the latter. This would either indicate that Pockels effect does not play a determinant role on the results or, on the other hand, that tensile stress would be a much more efficient way to enhance the Pockels effect.

As it was previously shown, there is a pre-existing positive fixed charge near the interface between nitride and silicon, which arise primarily from nitride and silicon dangling bonds (the so called N and K centers). The amount of this fixed charge and also the amount of interface traps can be strongly affected during annealing processes. Different studies have reported the passivation/depassivation of bulk defects due to hydrogen diffusion into the silicon nitride films after a heating process^34, 35^. Dangling bonds can be neutralized either by healing Si-N bonds or by an hydrogen atom, which creates new N-H and Si-H bonds and causes the decrease of the fixed charge in the film. The opposite, meaning a dehydrogenation of the silicon nitride layer, would lead to an increase of dangling bonds if no more Si-N bonds are created instead.

The strong influence of free-carriers is also confirmed in the electro-optic response measured after the annealing process. The effective index change for the sample with compressive stress (Fig. 5(b)) shows the characteristic peak and a completely analogous curve to that obtain in the simulations for lower concentrations of positive fixed charge (Fig. 1(c)), which arises during the annealing process. Although the existence of Pockels effect cannot be completely discarded, the obtained results strongly support that the measured effective index change is mainly originated due to the free carrier redistribution inside the waveguide. The evolution of the hydrogen content into the silicon nitride film during the heating process is found to be different depending on the deposition conditions and its composition^34, 35^. This is also reflected in the electro-optic results. While a clear change after the annealing can be observed in the sample with compressive stress, a linear behaviour is still found for the tensile stressed one (Fig. 5(c)), which means a lack of defect healing during the annealing in this case. In order to confirm the relationship between the reduction of fixed charge and defect passivation, Raman measurements have been carried out for both samples before and after the annealing. The spectral positions of the Raman peaks correspond to the vibrational frequencies of the molecules in the film. Position, area and width give information about atomic composition and bond arrangements.

**Figure 6:**
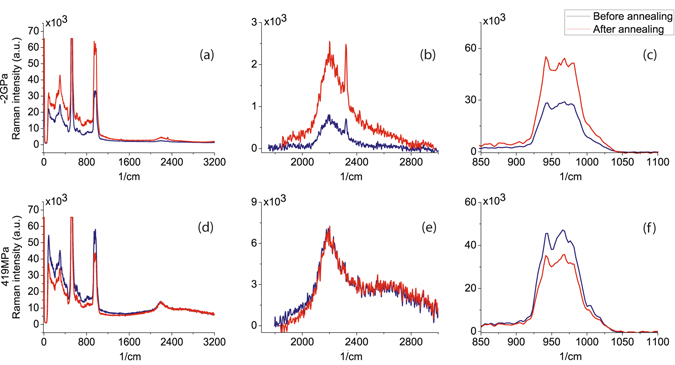
Raman spectra. Top Figures show Raman spectra obtained for the compressive stress sample, where (**a**) shows the complete Raman spectra and Figures (**b** and **c**) show a zoom on the range where peaks related to Si-H and Si-N bonds are located respectively. Analogously, (**d**) shows the complete Raman spectra for the 419 MPa sample and Figures (**e** and **f**) show a zoom on Si-H, Si-N related peaks.

Raman results are depicted in Fig. 6 where the peaks related to the Si-H and Si-N bonds have been zoomed for clarity. Peaks have been identified by means of a Lorentzian fitting. Area and peak position are shown in Table 2. Results show a great increase in the area of the Si-H and Si-N peaks for the compressive sample, especially the area of the Si-H(N_3_) bond has increased ~24 times, which indicates a film hydrogenation and experimentally confirms the initial hypothesis of a positive fixed charge decrease due to the heating process.

**Table 2:** Lorentzian fitting results for the compressive and tensile stress samples before and after the annealing process.

	Before annealing			After annealing		
	Vibration type	Centre (*cm* ^−1^)	Area (10^5^ *cm* ^−2^)	Centre (*cm* ^−1^)	Area (10^5^ *cm* ^−2^)	$\frac{{A}_{After}}{{A}_{Before}}$
Compressive	Si-H(*N*_2_Si)	2205.1	1.61	2188.6	2.81	1.74
	Si-H(*N*_3_)	2321.1	0.157	2301.1	3.75	23.9
	Si-N bending	972.1	18.6	972.4	34.2	1.84
	Si-N stretching 1	942.3	6.59	942.5	12.2	1.85
	Si-N streshing 2	822.9	4.59	822.5	10.1	2.20
Tensile	Si-H(*N*_2_Si)	2198.9	26.7	2198.8	25.8	0.97
	Si-H(*N*_*x*_)	2625.2	16.5	2620.5	25.1	1.52
	Si-N bending	970.4	30.1	971.2	22.1	0.74
	Si-N stretching 1	941.3	9.80	941.9	7.89	0.80
	Si-N streching 2	822.7	7.64	823.0	6.39	0.84

On the other hand, the reason why it was not possible to observe the peak in the tensile sample (Fig. 5(c)) is also confirmed by a general slight decrease in the hydrogen content of the film. It is possible to see in Table 2 that the area of the Si-N and Si-H bonds has remained almost constant or even decrease (with the exception of a small increase in Si-H(N_*x*_) bonds), which again supports the initial hypothesis. Finally, the order of magnitude of the effective index change remains the same after the annealing step for both samples although measurements show a difference in stress due to the annealing of −40% and +26% respectively for the compressive and tensile samples. We attribute the change in the hysteresis effect found for both samples before and after the annealing (Fig. 5(b,c)) to be related to the dynamics at the interface. A relationship between bulk and interface passivation is found in silicon nitride films^35^. In our results, it seems to be a correlation between higher hydrogenation/passivation and a stronger hysteresis. The time dependency found for the interface trap creation is quite slow and, therefore, an increase in the hydrogen bonds at this interface would slow the process of interface trap creation compared to a scenario where most of the dangling bonds are already created, giving rise to an increased hysteresis effect. This is what we observe for the compressive sample after the annealing while the opposite is obtained for the tensile sample, in agreement with the reduction of H revealed by Raman results. The influence of interface trap dynamics was also analyzed by measuring the electro-optical response for different time steps. Figure 5(d) shows the effective index change for the tensile sample after anneling taking into account a time step of 1, 3.5 and 10 minutes between each voltage measurement. It can also be seen that longer time steps give rise to smaller effective index changes due to the higher amount of interface traps created.

## Conclusion

In summary, the obtained results confirm that carrier effects can play a prominent role in the performance of strained silicon devices. We have demonstrated that the trapping properties at the interface between the silicon and silicon nitride have a strong influence on the electro-optic static response. The role played by the charge interchange with the silicon nitride film has also been recently suggested^24^. In that case, the transfer of positive charges from the silicon nitride film to the silicon waveguide was proposed to be the main origin of the electro-optic response. In our case, however, experimental results supported by simulations indicate that the silicon nitride charge is affected by the trapping dynamics at the interface resulting in a hysteretic response and the possibility of having saturation effects. In addition, the impact of the magnitude and nature of the applied stress has also been analyzed. Identical samples but with opposite tensile and compressive intrinsic stresses have been characterized. A further annealing was also carried out to change the magnitude of the applied stress. However, in all cases, we did not see any correlation between the electro-optic response and the applied stress that precludes a significant contribution of the Pockels effect. By contrary, the measured electro-optic response was consistent with variations of the silicon nitride fixed charge and the interface trapping properties, which was also confirmed by Raman measurements.

## Methods

### Simulations

Silvaco software was used to simulate the device performance. The influence of interface traps was simulated by using the Atlas package. Fermi-Dirac statistics and a concentration-dependent lifetime Shockley-Read-Hall generation-recombination model were used. The fundamental mode and associated effective index change and absorption losses due to free carriers was calculated by the Laser module. Stress distribution inside the structures was obtained using the Athena package specifying the silicon nitride intrinsic stresses experimentally measured. The strain and the figure of merit of eq. (2) was calculated with Matlab from the stress components and the electric field of the mode obtained with Silvaco.

### Fabrication

Devices were patterned on a silicon-on-insulator (SOI) wafer with a 220 nm top silicon layer and with a p-doping silicon concentration of 10^15^ *cm* ^−3^ and a buried oxide of 2 *μ*m. The deposition of the silicon nitride films was performed by using a parallel-plate PECVD (Applied Materials Centura 5200) at 400 °C with a 13.56 MHz RF generator and *NH*_3_ and *S*_*i*_ *H*_4_ as a gas phase precursors. Samples were annealed at 500 °C on a tubular furnace (Carbolite) during 30 minutes in atmospheric environment.

### Characterization

The silicon nitride intrinsic stress was characterized with a thin-film stress measurement instrument (Tencor model FLX- 5400) by measuring the wafer curvature before and after the annealing. Raman spectra were obtained with a confocal Raman spectroscopy equipment (Witec Alpha300RA model) with an excitation laser working at 532 nm and a spot size of 1 *μ*m. Scans were performed with a laser power of 39 mW and two accumulations of 30 seconds.

## References

[1] Reed GT, Mashanovich G, Gardes FY, Thomson DJ (2010). Silicon optical modulators. Nature Photonics.

[2] Rao A (2016). High-performance and linear thin-film lithium niobate Mach-Zehnder modulators on silicon up to 50 GHz. Optics Letters.

[3] Xiong C (2014). Active silicon integrated nanophotonics: ferroelectric *BaT*_*i*_*O*_3_ devices. Nano Letters.

[4] Castera P, Tulli D, Gutierrez AM, Sanchis P (2015). Influence of *BaT*_*i*_*O*_3_ ferroelectric orientation for electro-optic modulation on silicon. Optics Express.

[5] Melikyan, A. *et al*. High-speed plasmonic phase modulators. *Nature Photonics* 5–9 (2014).

[6] Jacobsen RS (2006). Strained silicon as a new electro-optic material. Nature.

[7] Hon NK, Tsia KK, Solli DR, Jalali B (2009). Periodically poled silicon. Applied Physics Letters.

[8] Chmielak B (2011). Pockels effect based fully integrated, strained silicon electro-optic modulator. Optics Express.

[9] Avrutsky I, Soref R (2011). Phase-matched sum frequency generation in strained silicon waveguides using their second-order nonlinear optical susceptibility. Optics Express.

[10] Schriever C, Bohley C, Schilling J, Wehrspohn RB (2012). Strained Silicon Photonics. Materials.

[11] Bianco F (2012). Two-dimensional micro-Raman mapping of stress and strain distributions in strained silicon waveguides. Semiconductor Science and Technology.

[12] Chmielak B (2013). Investigation of local strain distribution and linear electro-optic effect in strained silicon waveguides. Optics Express.

[13] Aleali, A., Xu, D., Schmid, J. H., Cheben, P. & Winnie, N. Y. Optimization of stress-induced pockels effect in silicon waveguides for optical modulators. In *Group IV Photonics (GFP), 2013 IEEE 10th International Conference on*, 109–110 (IEEE, 2013).

[14] Puckett MW, Smalley JS, Abashin M, Grieco A, Fainman Y (2014). Tensor of the second-order nonlinear susceptibility in asymmetrically strained silicon waveguides: analysis and experimental validation. Optics Letters.

[15] Damas P (2014). Wavelength dependence of pockels effect in strained silicon waveguides. Optics Express.

[16] Khurgin JB, Stievater TH, Pruessner MW, Rabinovich WS (2015). On the origin of the second-order nonlinearity in strained Si-SiN structures. JOSA B.

[17] Manganelli CL, Pintus P, Bonati C (2015). Modeling of strain-induced Pockels effect in Silicon. Optics Express.

[18] Damas P, Marris-Morini D, Cassan E, Vivien L (2016). Bond orbital description of the strain-induced second-order optical susceptibility in silicon. Physical Review B.

[19] Cazzanelli M (2012). Second-harmonic generation in silicon waveguides strained by silicon nitride. Nature Materials.

[20] Schriever C (2015). Second-Order Optical Nonlinearity in Silicon Waveguides: Inhomogeneous Stress and Interfaces. Advanced Optical Materials.

[21] Borghi MB (2015). High-frequency electro-optic measurement of strained silicon racetrack resonators. Optics Letters.

[22] Azadeh SS, Merget F, Nezhad M, Witzens J (2015). On the measurement of the Pockels effect in strained silicon. Optics Letters.

[23] Sharma R (2016). Effect of dielectric claddings on the electro-optic behavior of silicon waveguides. Optics Letters.

[24] Borghi M (2016). Homodyne Detection of Free Carrier Induced Electro-Optic Modulation in Strained Silicon Resonators. Journal of Lightwave Technology.

[25] Olivares, I., Ivanova, T., Pinilla-Cienfuegos, E. & Sanchis, P. A systematic optimization of design parameters in strained silicon waveguides to further enhance the linear electro-optic effect. In *SPIE Photonics Europe*, 98910E-98910E (International Society for Optics and Photonics, 2016).

[26] Warren WL, Lenahan P, Curry SE (1990). First observation of paramagnetic nitrogen dangling-bond centers in silicon nitride. Physical review letters.

[27] Warren W, Kanicki J, Robertson J, Poindexter E, McWhorter P (1993). Electron paramagnetic resonance investigation of charge trapping centers in amorphous silicon nitride films. Journal of applied physics.

[28] Bazilchuk M, Haug H, Marstein ES (2015). Modulating the fixed charge density in silicon nitride films while monitoring the surface recombination velocity by photoluminescence imaging. Applied Physics Letters.

[29] Stathis JH, Zafar S (2006). The negative bias temperature instability in MOS devices: A review. Microelectronics Reliability.

[30] Alam MA, Mahapatra S (2005). A comprehensive model of PMOS NBTI degradation. Microelectronics Reliability.

[31] Kufluoglu H, Alam MA (2004). A computational model of NBTI and hot carrier injection time-exponents for MOSFET reliability. Journal of Computational Electronics.

[32] Schmidt J, Schuurmans FM, Sinke WC, Glunz SW, Aberle AG (1997). Observation of multiple defect states at silicon-silicon nitride interfaces fabricated by low-frequency plasma-enhanced chemical vapor deposition. Applied Physics Letters.

[33] Sanjoh A, Ikeda N, Komaki K, Shintani A (1990). Analysis of Interface States between Plasma-CVD Silicon Nitride and Silicon-Substrate Using Deep-Level Transient Spectroscopy. Journal of The Electrochemical Society.

[34] Martnez F, Mártil I, González-Daz G, Selle B, Sieber I (1998). Influence of rapid thermal annealing processes on the properties of SiN_*x*_:H films deposited by the electron cyclotron resonance method. Journal of non-crystalline solids.

[35] Martnez F (1999). Thermal stability of a-SiN_*x*_:H films deposited by plasma electron cyclotron resonance. Journal of Vacuum Science & Technology A: Vacuum, Surfaces, and Films.

